# Supplementary material for: MeCP2 regulates Gdf11, a dosage-sensitive gene critical for neurological function
Source: eLife. 2023 Feb 27;12:e83806. doi: 10.7554/eLife.83806 (PMC9977283; doi:10.7554/eLife.83806)
Supplement: Supplementary file 1. [file elife-83806-supp1.docx]

**Supplementary File 1. Summary and source of transcriptomics studies mined in this study.**

| **Study no.^a^** | **Model and brain region** | **Organism** | **Measurement platform** | **Data source** | **Citation** |
| --- | --- | --- | --- | --- | --- |
| 1 | Postmortem RTT excitatory neurons | Human | Single nuclei RNA-seq | Supplemental Table 7 | (Renthal et al., 2018) |
| 2 | Postmortem RTT VIP neurons | Human | Single nuclei RNA-seq | Supplemental Table 8 | (Renthal et al., 2018) |
| 3 | *Mecp2^-/y^* cerebellum | Mouse | Bulk RNA-seq | Re-analyzed from counts deposited in GSE105045 | (Raman et al., 2018) |
| 4 | *Mecp2^-/y^* cortex | Mouse | Bulk RNA-seq | Supplemental Table S1 | (Boxer et al., 2020) |
| 5 | *Mecp2^-/y^* cortex | Mouse | Bulk RNA-seq | Supplemental Data Set S03 | (Zhou et al., 2022) |
| 6 | *Mecp2^-/y^* cortex | Mouse | Bulk RNA-seq | Supplemental Table S1 | (Clemens et al., 2020) |
| 7 | *Mecp2^-/y^* cortex | Mouse | Bulk RNA-seq | Analyzed table deposited in GSE142716 | (Jiang et al., 2021) |
| 8 | *Mecp2^-/y^* dentate gyrus | Mouse | Bulk RNA-seq | Figure 5 – Source data 1 | (Pohodich et al., 2018) |
| 9 | *Mecp2^-/y^* hypothalamus | Mouse | Bulk RNA-seq | <https://zenodo.org/record/1226607#.YyFCwC-B0lV> ; (Raman et al., 2018) | (Chen et al., 2015) |
| 10 | *Mecp2^-/y^* hypothalamus | Rat | Bulk RNA-seq | Re-analyzed from counts deposited in GSE83323 | (Veeraragavan et al., 2016) |
| 11 | *Mecp2^T158M/y^* cortex | Mouse | Bulk RNA-seq | Analyzed table deposited in GSE142716 | (Jiang et al., 2021) |
| 12 | *Mecp2^R306C/y^* cortex | Mouse | Bulk RNA-seq | Supplemental Table S1 | (Boxer et al., 2020) |
| 13 | *Mecp2^R270X/y^* hippocampus | Mouse | Microarray | <https://zenodo.org/record/1226607#.YyFCwC-B0lV> ; 9-week experiment; (Raman et al., 2018) | (Baker et al., 2013) |
| 14 | *Mecp2^G273X/y^* hippocampus | Mouse | Microarray | <https://zenodo.org/record/1226607#.YyFCwC-B0lV> ; 9-week experiment; (Raman et al., 2018) | (Baker et al., 2013) |
| 15 | *Mecp2^+/-^* dentate gyrus | Mouse | Bulk RNA-seq | Figure 6 – Source data 1 | (Pohodich et al., 2018) |
| 16 | *MECP2* duplication hippocampus | Mouse | Bulk RNA-seq | Re-analyzed from counts deposited in GSE71229 ; genetic rescue experiment | (Sztainberg et al., 2015) |
| 17 | *MECP2* duplication hippocampus | Mouse | Bulk RNA-seq | Re-analized from counts deposited in GSE151222 | (Shao et al., 2021b) |
| 18 | *MECP2* triplication hypothalamus | Mouse | Bulk RNA-seq | <https://zenodo.org/record/1226607#.YyFCwC-B0lV> ; (Raman et al., 2018) | (Chen et al., 2015) |
| 19 | *MECP2* triplication cortex | Mouse | Bulk RNA-seq | Supplemental Table S1 | (Clemens et al., 2020) |
| 20 | *MECP2* AAV expressing sorted neurons | Mouse | Sorted nuclear RNA-seq | Extended Data Fig. 12-1; High population only | (Ito-Ishida et al., 2020) |

**^a^** Refers to the order of studies in Figure 1B and Figure 1-supplemental figure 1B-D
